# Short communication: genetic variations of *SLC2A9* in relation to Parkinson’s disease

**DOI:** 10.1186/2047-9158-2-5

**Published:** 2013-02-19

**Authors:** Jianjun Gao, Hong Xu, Xuemei Huang, Honglei Chen

**Affiliations:** 1 Epidemiology Branch, National Institute of Environmental Health Sciences, Research Triangle Park, North Carolina, USA; 2 Molecular Genetics Core Facility, National Institute of Environmental Health Sciences, Research Triangle Park, North Carolina, USA; 3 Department of Neurology, Pennsylvania State University-Milton S. Hershey Medical Center, Hershey, Pennsylvania, USA

## Abstract

**Background:**

Epidemiological studies showed that higher plasma urate was associated with lower risk for Parkinson’s disease (PD) and slower disease progression. Recent genome-wide association studies (GWAS) consistently showed that several single nucleotide polymorphisms (SNPs) in the solute carrier family 2 member 9 gene (*SLC2A9* ) were associated with plasma urate concentration and the risk of gout.

**Methods:**

We conducted a case–control study to examine twelve tag SNPs of the *SLC2A9* gene in relation to PD among 788 cases and 911 controls of European ancestry. Odds ratios (OR) and 95% confidence intervals (CI) were derived from logistic regression models, adjusting for age, sex, smoking and caffeine consumption.

**Results:**

These SNPs were all in linkage disequilibrium (R^2^ > 0.7). None of them were associated with PD risk. Among women, however, there was a suggestion that the presence of the minor allele of one SNP (rs7442295) was related to a small increase in PD risk [OR (95% CI) = 1.48 (1.01-2.16)].

**Conclusion:**

This study provides little support for genetic variations of *SLC2A9* and PD risk.

## 

Parkinson’s disease (PD) is the second most prevalent neurodegenerative disease and the causes are largely unknown. It has been long hypothesized that, during PD development, oxidative stress contributes to the loss of dopaminergic neurons in the substantia nigra. Uric acid is a potent endogenous antioxidant that effectively scavenges reactive nitrogen and oxygen radicals, and thus may protect against PD. Data from large observational studies consistently showed that higher plasma urate predicted a lower risk of PD [1,2] and slower disease progression [3]. The nature of this association is however debatable as one may argue that the association is due to reverse causation that lower plasma urate is secondary to the underlying PD pathogenesis. Investigations into urate genetics may help clarify this observation. For example, by examining the association of urate related genetic alleles with PD, we may have an unbiased assessment of urate in relation to PD risk to the extent that these genetic alleles affect plasma urate, a concept called “Mendelian randomization” [4]. Recent genome-wide association studies (GWAS) consistently showed that the minor alleles of several single nucleotide polymorphisms (SNPs) in the solute carrier family 2 member 9 gene (*SLC2A9* ) were associated with lower plasma urate concentration and predicted lower gout risk [5,6]. We therefore examined these genetic variations in relation to PD risk in a case–control study of 788 PD cases and 911 controls, all non-Hispanic Caucasians.

## Methods

Details of the study method were published previously [7]. Briefly, we first identified self-reported PD cases from a large population-based cohort – the NIH-AARP Diet and Health Study. We then asked the treating physicians to fill out a short diagnostic questionnaire and to send a copy of relevant medical records. The diagnoses were either confirmed by their treating neurologists or via medical record review by a movement disorder specialist (XH). Only confirmed PD patients were included in the present analyses. Controls were randomly selected from participants of the same cohort who did not report a PD diagnosis and they were frequency matched to cases by sex, ethnicity, and year of birth. Therefore, both cases and controls were from the same base population and no additional exclusion criteria were applied. Both cases and controls provided saliva samples from which DNAs were extracted. Twelve SNPs were selected from *SLC2A9* based on recent GWAS findings on plasma urate [5,6]. Genotyping was conducted using the Illumina BeadXpress platform with a call rate of 99.9%. All participants provided written consent and the study protocol was approved by relevant institutional review boards. To avoid population stratification, the current analysis was limited to 788 cases and 911 controls who self-reported as non-Hispanic Whites.

Hardy-Weinberg equilibrium among controls was confirmed with chi-square statistics for all SNPs (*P* > 0.05). Odds ratios (ORs) and 95% confidence intervals (CIs) were derived from logistic regression models under the assumption of logit-additive allelic effects, adjusting for age, sex, smoking status and caffeine intake. All statistical analyses were performed using SAS version 9.2 (SAS Institute Inc, Cary, NC), and two sided p < 0.05 was considered statistically significant. We performed power calculations with Quanto version 1.2.4.

## Results

Table 1 showed population characteristics of PD patients and controls. Cases and controls were matched by birth year in five year groups and gender. For cases, the average age of PD diagnosis was 66.6 ± 7.4 years and the average age of PD onset was 65.7 ± 7.4 years. As expected, cases were less likely to smoke and had lower caffeine intake than controls.

**Table 1:** Population characteristics of cases and controls*

	**Control (%)**	**Case (%)**
N	911	788
Gender		
Men	725 (79.58)	606 (76.90)
Women	186 (20.42)	182 (23.10)
Age at baseline, years	63.5 ± 4.8	63.0 ± 5.0
Smoking status		
Never	318 (34.91)	363 (46.07)
Former	540 (59.28)	391 (49.62)
Current	49 (5.38)	23 (2.92)
Caffeine intake, mg/day	364.91 ± 362.26	295.54 ± 334.69
Age at onset, year		65.73 ± 7.43
Age at diagnosis, year		66.65 ± 7.37

* Numbers may not add up to total due to missing.

The 12 SNPs from *SLC2A9* were all in linkage disequilibrium (R^2^ > 0.7). In general, none of these SNPs were associated with PD in overall and gender specific analyses (Table 2). Among women, there was a suggestion that the presence of the minor allele of one *SLC2A9* SNP (rs7442295) was related to a small increase in PD risk (OR = 1.48, 95%CI = 1.01-2.16). The direction of this relation is consistent with that would be predicted based on the relationship of this SNP to plasma urate. The association was however not statistical significant after adjusting for multiple comparisons.

**Table 2:** Selected *SLC2A9 *SNPs in relation to the risk of Parkinson’s disease*

	**All**				**Men**				**Women**			
**SNPs**	**MAF**		**OR (95% CI)**	***P***	**MAF**		**OR (95% CI)**	***P***	**MAF**		**OR (95% CI)**	***P***
	Case	Control			Case	Control			Case	Control		
rs16890979	0.23	0.22	1.06 (0.90-1.24)	0.51	0.22	0.23	0.99 (0.82-1.19)	0.92	0.23	0.18	1.40 (0.96-2.03)	0.08
rs13129697	0.26	0.27	0.99 (0.85-1.16)	0.91	0.26	0.27	0.95 (0.79-1.13)	0.53	0.28	0.24	1.19 (0.84-1.69)	0.33
rs737267	0.25	0.25	1.01 (0.86-1.18)	0.91	0.25	0.25	0.97 (0.81-1.16)	0.70	0.25	0.22	1.20 (0.85-1.70)	0.30
rs6855911	0.25	0.25	1.00 (0.85-1.18)	0.98	0.24	0.25	0.95 (0.80-1.14)	0.61	0.25	0.22	1.21 (0.85-1.71)	0.29
rs4697700	0.24	0.23	1.03 (0.88-1.22)	0.69	0.24	0.24	1.02 (0.85-1.22)	0.87	0.23	0.21	1.13 (0.79-1.62)	0.49
rs4481233	0.19	0.19	1.01 (0.85-1.21)	0.90	0.19	0.20	0.96 (0.79-1.18)	0.72	0.19	0.16	1.25 (0.84-1.87)	0.28
rs7442295	0.22	0.21	1.06 (0.90-1.26)	0.50	0.22	0.22	0.98 (0.81-1.19)	0.83	0.23	0.17	1.48 (1.01-2.16)	0.04
rs6449213	0.19	0.19	1.01 (0.85-1.21)	0.88	0.20	0.20	0.97 (0.79-1.18)	0.74	0.19	0.16	1.23 (0.83-1.84)	0.31
rs1014290	0.25	0.25	1.04 (0.88-1.22)	0.67	0.26	0.25	1.07 (0.89-1.28)	0.48	0.23	0.24	0.91 (0.63-1.31)	0.62
rs12509955	0.21	0.22	0.99 (0.84-1.18)	0.93	0.22	0.22	1.00 (0.82-1.20)	0.96	0.19	0.20	0.99 (0.67-1.46)	0.95
rs17251963	0.21	0.20	1.05 (0.88-1.25)	0.59	0.21	0.20	1.02 (0.84-1.24)	0.83	0.20	0.18	1.17 (0.78-1.75)	0.44
rs12510549	0.21	0.22	0.96 (0.81-1.14)	0.65	0.21	0.23	0.91 (0.76-1.10)	0.34	0.20	0.18	1.22 (0.83-1.79)	0.31

* Odds ratios and 95% confidence intervals were derived from logistic regression models under the assumption of logit-additive allelic effects, adjusting for age, sex, smoking status and caffeine intake.

MAF: minor allele frequency; OR: odds ratio; CI: confidence intervals.

## Discussion

PD has an insidious onset and a long latency period, therefore epidemiological findings are often subject to competing explanations of biology versus reverse causality that the exposures are secondary to the underlying disease process. Although the exact biological mech-anisms are yet to be revealed, higher plasma urate has been linked to lower PD risk in several prospective studies [1,2]. Plasma urate increases with age in human and has been hypothesized to protect dopaminergic neurons via its potent antioxidant properties. On the other hand, one cannot exclude the possibility that plasma urate rises in response to increased free radicals as a result of PD pathogenesis.

Genetic analyses may help to clarify such epidemiologic findings. Unlikely diet, lifestyle or many other environmental exposures, genetic variants are fixed and not affected by disease process. They are randomly assorted during gamete formation in a process that is similar to randomization in clinical trials. “Mendelian randomization” has thus been proposed to investigate causality of epidemiologic findings by circumventing reverse causation and confounding [4]. If a non-genetic exposure is causally related to an outcome, robust genetic determinants of this exposure are expected to relate to the outcome. Previous Mendelian randomization analyses using large samples support a causative relationship of plasma urate with gout, but argue against one with cardiovascular diseases [8,9].

To the best of our knowledge, this is the first study to follow up recent GWAS finding on *SLC2A9* and plasma urate for PD research, and some of these SNPs were included in the above-mentioned studies on gout and cardiovascular diseases [8,9]. *SLC2A9* was the first urate transporter gene that was identified from GWAS and has shown the most robust association with plasma urate and gout in previous studies [8,9]. Reduced renal excretion of urate is the most important cause of high plasma urate and gout, and *SLC2A9* encodes a major urate transporter [10]. Although the exact causal variants of the *SLC2A9* gene that determine plasma urate are yet to be identified, GWAS studies have linked several tag SNPs in this gene to the level of plasma urate. However, in GWAS, these SNPs only explain a small amount of plasma urate concentration [5] and thus the expected OR for PD associated with these SNPs are smaller than 1.1 for both men and women. Although our study is among the largest population-based studies on PD with both genetic and environmental data, the power of our study to detect such weak associations was limited. With 788 cases and 911 controls, we estimated that we had 80% power to identify an OR of higher than 1.25 or lower than 0.79 for a minor allele frequency of 25%. Other limitations of the current study include the lack of plasma urate, and that we did not include genetic variations from other key genes that are related to plasma urate as identified in later GWAS studies [11].

In summary, our analysis did not support a relationship between these *SLC2A9* SNPs and PD in men, but it does not exclude a modest association for women.

## Competing interests

The authors declare that they have no competing interests.

## Authors’ contributions

JG contributed to study concept, conducted data analysis, and drafted the manuscript. HX and XH contributed to data collection and provided critical comments. HC contributed to data collection, study concept, and manuscript preparation and oversaw this project. All authors read and approved the final manuscript.

## Financial disclosure

None reported.

## Funding/Support

This study was supported by the intramural research program of the NIH, the National Institute of Environmental Health Sciences (Z01-ES-101986), and NIH extramural grant to Dr. Huang (NS06722).
